# Pretreatment haemoglobin levels significantly predict the tumour response to primary chemotherapy in human breast cancer

**DOI:** 10.1038/sj.bjc.6601216

**Published:** 2003-09-09

**Authors:** A Bottini, A Berruti, M P Brizzi, A Bersiga, D Generali, G Allevi, S Aguggini, G Bolsi, S Bonardi, G Bertoli, P Alquati, L Dogliotti

**Affiliations:** 1 Breast Unit, Azienda Ospedaliera Istituti Ospitalieri, Cremona, Italy; 2 Dipartimento di Scienze Cliniche e Biologiche, Università di Torino, Oncologia Medica, Azienda Ospedaliera San Luigi, Regione Gonzole 10 Orbassano, 10043, Italy; 3 Anatomia Patologica, Azienda Ospedaliera Istituti Ospitalieri, Cremona, Italy

**Keywords:** breast cancer, primary chemotherapy, haemoglobin, predictive factors, tumour response

## Abstract

The purpose of this study was to evaluate whether tumour response to primary chemotherapy in human breast cancer is influenced by baseline haemoglobin (Hb) status. A total of 157 patients with T2-4, N0-1 M0 breast cancer were treated with chemotherapy consisting of either the CMF regimen + tamoxifen (the first 76 cases) or the single-agent epirubicin (the subsequent 81) before definitive surgery. In total, 144 patients were fully assessable. Ki67, p53, bcl-2, c-erbB2, steroid hormone receptor, and microvessel density were evaluated immunohistochemically in tumour specimens obtained before chemotherapy and at surgery. Tumour shrinkage >50% occurred in 72.1% of patients. Responding patients had higher baseline Hb levels and red blood cell counts than nonresponders (*P*<0.01 and <0.003, respectively). The distribution of disease response according to increasing cutoffs of baseline Hb status showed that from 12.5 mg l^−1^ onwards, patients with Hb levels above the cutoff obtained a greater response rate than those with lower Hb values. The difference attained the statistical significance at 12.5 (76.1 *vs* 59.5%, *P*<0.05) and 13.0 g/dl^−1^ (81.0 *vs* 57.6%, *P*<0.002) cutoffs, respectively. The predictive role of Hb levels was maintained in multivariate analysis after adjustment for clinical and biological characteristics and treatment regimen. Patients with baseline Hb levels ⩽13 g dl^−1^ showed a lower treatment-induced reduction in Ki67 expression (*P*<0.04) and a higher Ki67 expression at postoperative evaluation (*P*<0.02) than their counterparts. In conclusion, low Hb levels may negatively influence the response rate of chemotherapy in breast cancer patients. Inhibition of antiproliferative activity could be a possible mechanism.

Tumour hypoxia has been shown to play an important role in the outcome of cancer patients. Low intratumoural oxygen levels may select for a more malignant phenotype (Coleman *et al*, 2001; Feldmann, 2001; Hockel and Vaupel, 2001) and provoke tumour aggressiveness, with a greater likelihood of malignant progression and decreased responsiveness to standard therapy (Coleman *et al*, 2001; Feldmann, 2001; Hockel and Vaupel, 2001; Wouters *et al*, 2002).

Hypoxia is able to promote tumour metastasis by inducing the expression of gene products involved in the metastatic cascade (Graham *et al*, 1999; Xu *et al*, 1999; Canning *et al*, 2001), and by stimulating neoangiogenesis (Semenza, 2001). Also, hypoxia may mediate a selection pressure for a more aggressive phenotype by promoting the clonal expansion of cells with diminished apoptotic potential (Graeber *et al*, 1996).

Traditionally, Tumour hypoxia is considered a potential therapeutic problem since it renders solid tumours more resistant to radiation therapy (Feldmann, 2001; Hockel and Vaupel, 2001). Moreover, under hypoxic conditions, the fraction of proliferating tumour cells has been found to decrease, thus compromising the effectiveness of most chemotherapeutic drugs that are primarily effective against rapidly dividing cells (Eisenhauer *et al*, 1997; Green and Giaccia, 1998).

The oxygenation status of human tumours is mainly dependent on (1) the availability of oxygen via the blood, and (2) the diffusional flux from the microvessel to the oxygen-consuming cell. While oxygen availability process is a function of haemoglobin (Hb) blood concentration, diffusional flux depends mainly on the *p*O_2_ gradient and on the diffusion distance between vessels and tumour cells (Coleman *et al*, 2001; Feldmann, 2001; Hockel and Vaupel, 2001; Wouters *et al*, 2002).

Haemoglobin concentration has been shown to be an important predictive factor for the outcome of radiotherapy or chemo-radiotherapy in the treatment of various types of cancer, including uterine – cervix cancer (Fyles *et al*, 1998), head and neck cancer (Glaser *et al*, 2001) and bronchogenic carcinoma (Kovacs *et al* 1998). Data on the predictive role of Hb on disease response to chemotherapy are lacking.

Preoperative chemotherapy administered to breast cancer patients is the best model to identify baseline features able to predict which patients may be most likely to benefit or not from cytotoxic treatment (Bonadonna *et al*, 1998; Mamounas and Fisher, 2001). This treatment modality allows a perfectly quantifiable evaluation of the chemosensitivity or chemoresistance of any treated case. In addition, tumour specimen biopsy obtained in matched pair cases at diagnosis and definitive surgery can provide valuable information on treatment-induced changes in tumour biology (Fisher and Mamounas, 1995; Bottini *et al*, 2000).

In this study, we evaluated baseline Hb levels as a predictive factor for response to treatment in a consecutive series of breast cancer patients receiving primary chemotherapy in a single institution.

The primary aim of the study was to investigate the effect of baseline Hb values on tumour shrinkage induced by the treatment. Secondary aims were: (1) to correlate Hb values with biological parameters and (2) to correlate Hb values with changes in Ki67 expression before and after treatment.

## PATIENTS AND METHODS

### Patients

From August 1990 to January 1997, 157 consecutive patients with an operable breast tumour or locally advanced disease (T2-4, N0-1, M0) had been enrolled in two consecutive phase II studies that tested the activity of the CMF regimen (cyclophosphamide, methotrexate, 5-fluorouracil) combined with tamoxifen and the activity of the single-agent epirubicin. None of the patients had objective skin inflammation or Oedema. On first presentation, an incision biopsy was performed on each patient. Initial staging comprised clinical examination, bilateral mammography, echography, chest X-ray, liver echography or CT scan, bone scintigraphy. The study was approved by the institutional Investigations Committee. All patients gave written informed consent to the diagnostic procedures and the proposed treatment.

### Treatment

Chemotherapy was started within 1 or 2 days from diagnosis. The first consecutive 76 patients received the CMF chemotherapy regimen, which was given on days 1 and 8 every 28 days. The dose of cyclophosphamide and 5-fluorouracil was 600 mg m^−2^ of body surface area, and the dose of methotrexate was 40 mg m^−2^. The subsequent 81 patients received epirubicin 60 mg m^−2^ on days 1 and 2 every 21 days. The first consecutive 45 patients with oestrogen-positive (ER+) breast cancer at first biopsy received additional tamoxifen (TAM) (30 mg daily) in association with the CMF treatment. Each month, the size of the primary tumour and the size of the axillary lymph nodes, when appreciable, were carefully measured with a caliper by the same clinician. Response was assessed by clinical measurement of the changes in the product of the two largest diameters recorded in two successive evaluations. Based on World Health Organization (WHO) criteria (Miller *et al*, 1981), tumour progression (PD) was defined as an increase of at least 25% in tumour size, stable disease (SD) as an increase of less than 25% or a reduction of less than 50%, partial response (PR) as a tumour shrinkage greater than 50%, and complete response (CR) as the complete disappearance of all clinical signs of disease.

Surgery was planned after full clinical reassessment about 1 month after the end of the last chemotherapy cycle. Quadrantectomy or modified radical mastectomy was performed when indicated in association with full axillary dissection. All patients subjected to quadrantectomy underwent irradiation of the residual breast (60 Gy delivered over 6 weeks). Before and during chemotherapy, blood cell count was repeatedly evaluated; only those evaluations performed a few days before incisional biopsy and immediately before definite surgery were considered in the present analysis.

### Histopathologic grade and immunohistochemistry

The degree of malignancy was assessed according to the Elston and Ellis (1991) grading system. The immunohistochemical methodology used in this study is fully described elsewhere (Bottini *et al*, 2000). Briefly, an antigen-retrieval step was performed by heating a tissue section in a citrate buffer. The primary antibodies applied were: CD34/QB-END (mouse monoclonal (Novocastra Lab, Newcastle upon Tyne, UK), dilution 1 : 25, overnight incubation at 4°C); ER (mouse monoclonal 6F11 (Novocastra Lab), dilution 1 : 50, 1 h incubation at room temperature (RT); PgR (mouse monoclonal 1A6 (Novocastra Lab), dilution 1 : 20, 1 h incubation at RT); Ki67 (mouse monoclonal Mib-1 (Dako, Glostrup, Denmark), dilution 1 : 30, 1 h incubation at RT); p53 (mouse monoclonal D07 (Novocastra Lab), dilution 1 : 100, 1 h incubation at RT); bcl-2 (mouse monoclonal 124 (Dako), dilution 1 : 40, overnight at 4°C); and (c-erbB2 (mouse monoclonal CB11 (Novocastra Lab), overnight at 4°C).

Biotinylated horse anti-mouse IgG and avidin–biotin–peroxidase complex were applied as a staining method (Vectastatin ABC kit; Vector Laboratories, Inc., Burlingame, CA, USA). A solution containing hydrogen peroxide (0.06% v v^−1^) and diamino-benzidine 4 HCl (DAB; 0.05 v v^−1^) was used as chromogen.

### Immunohistochemical scoring

All samples had a negative control slide (no primary antibody) of an adjacent section to assess the degree of nonspecific staining. Positive controls included breast carcinomas known to exhibit high levels of each marker.

Staining was scored by counting the number of positive-stained cells and expressed as a percentage of the total tumour cells (at least 1000) counted across several representative fields of the section using a standard light microscope equipped with a 10 × 10 square graticule. Reproducibility of counting was assessed by a second investigator rescoring 10 slides.

Vascularity was defined as previously described (Bottini *et al*, 2002) as the number of vessels per field counted in the area of highest vascular density (‘hot spots’) at × 400 magnification. This corresponded to a field size of 0.43 mm^2^. Five fields per tumour section were counted in the areas that appeared to contain the greatest number of microvessels. The average of counts in these fields was considered in the analysis. All figures in the text are quoted per mm^2^.

The relative intensity of ER and PgR staining was assessed in a semiquantitative manner, as previously described by McCarty *et al* (1985), incorporating both the intensity and distribution of specific staining. A value (HSCORE) was derived from the sum of the percentages of positive-stained epithelial cells multiplied by the weighted intensity of staining. Specimens were deemed receptor positive if the HSCORE was greater than 100. For the other biological parameters, a cutoff of ⩾5 positive cells was introduced to discriminate p53-positive and p53-negative primary malignancies, as previously reported (Silvestrini *et al*, 1993). A cutoff of 10% stained cells was considered for c-erbB2 positivity. No cutoffs were introduced for bcl2 expression. The immunohistochemical evaluation at definitive surgery was performed by the same pathologists who remained blinded to the disease response and the score assessed at first biopsy.

### Statistical analysis

Parametric and nonparametric statistical methods (Student's *t*-test for paired and unpaired data, Mann–Whitney *U*-test for unpaired data, and *ρ* for simple correlation analysis) were used, when indicated, in the primary analyses of the data. The association between dichotomous variables was assessed by *χ*^2^. Multivariate analysis was performed by logistic regression. Relapse-free survival (RFS) and overall survival (OS) were calculated from diagnosis to the occurrence of relapse of disease or disease-related death. The OS and RFS curves were estimated using the Kaplan–Meier method. Unadjusted differences in these estimates between treatment groups were assessed with the log-rank test. Statistical analysis was performed on an IBM-compatible personal computer using the Statistica for Windows (Tulsa, OK, USA) software package (Statsoft Inc., 1995).

## RESULTS

One patient refused to continue the treatment plan at the end of the first cycle. Two patients with baseline Hb level of 8 g dl^−1^ were excluded from the study because they were bearing thalassemia minor. In total, 154 patients were therefore fully evaluable and included in the analysis. A median of three chemotherapy cycles was administered (range 3–5). Patient characteristics, treatment outcome and changes in immunohistochemical markers in the present series are described elsewhere (Bottini *et al*, 2000, 2001, 2002). Briefly, according to WHO criteria (Miller *et al*, 1981), 38 patients attained a clinical CR, 73 a PR, for an overall response rate of 72.1%. In all, 40 patients showed disease stabilization and three progressed. Three patients obtained a complete pathological response to treatment. Median Hb levels and red blood cell count (RBC) (range) were 13.5 g dl^−1^ (10.7–16.3) and 4.5 × 10^6^ *μ*l (3.5–6.0) at baseline and 10.8 g dl^−1^ (7.2–14.5) and 3.5 × 10^6^ *μ*l (2.4–5.4) before definitive surgery (*P*<0.001). There was a moderate correlation between baseline Hb and Hb evaluated before surgery (Spearman *r*=0.40, *P*<0.05). A similar relationship was observed for RBC (*r*=0.51, *P*<0.05). The decrease in Hb levels from baseline was greater in the subset of patients treated with the single-agent epirubicin than in the subgroup that received the CMF regimen (Table 1

**Table 1:** Haemoglobin (Hb) values before and after treatment according to cytotoxic drug administration

	**CMF±tamoxifen**	**Epirubicin**
*Baseline Hb*		
Mean (s.d.)	13.6 (1.11)	13.2 (1.0)
Median (range)	13.9 (10.7–16.0)	13.1 (10.9–16.3)

*Reduction Hb*		
Mean (s.d.)	−2.03 (1.30)	−3.13 (1.27)
Median (range)	−1.80 (−5.3–0.7)	−3.10 (−6.3–−0.3)

*Postchemotherapy Hb*		
Mean (s.d.)	11.6 (1.28)	10.1 (1.10)
Median (range)	11.5 (8.4–14.5)	10.1 (7.2–11.8)

CMF=cyclophosphamide, methotrexate, 5-fluorouracil; s.d.=standard deviation.

).

### Haemoglobin levels and treatment outcome

Haemoglobin and RBC at baseline condition in the 111 patients attaining a disease response (median 13.7 g dl^−1.^ (range 10.7–16.3) and 4.56 × 10^6^ *μ*l (3.7–6.0)) were higher than in those who did not (median 12.8 g dl^−1^ (range 11.2–16.0) and 4.29 × 10^6^ *μ*l (3.5–5.2)); *P*<0.01 and <0.003, respectively.

The distribution of disease response according to increasing cutoffs of baseline Hb status is outlined in Table 2

**Table 2:** Disease response to primary chemotherapy according to progressively higher cutoff of baseline haemoglobin (Hb) levels

Hb cutoffs mg dl^−1^	12.0	12.5	13.0	13.5	14.0

	Response rates				
Hb⩽the cutoff	9/12 (75.0%)	22/37 (59.5%)	34/59 (57.6%)	51/78 (65.4%)	77/112 (68.7%)
Hb>the cutoff	102/142 (71.8%)	89/117 (76.1%)	77/95 (81.0%)	60/76 (78.9%)	34/42 (80.9%)
*P*	=0.8	=0.05	<0.002	=0.06	=0.1

. From 12.5 mg l^−1^ onwards, patients with Hb levels above the cutoff obtained a greater response rate than those with lower Hb values. This difference attained statistical significance at 12.5 (*P*=0.05) and 13.0 g dl^−1^ (*P*<0.002). The cutoff of 13.0 g dl^−1^ was used for subsequent statistical computations. Accordingly, 95 patients had Hb values >13 g dl^−1^ and 59 had Hb values <13 g dl^−1^ on baseline conditions, while the corresponding distribution of patients at the end of treatment was 12 and 142, respectively. All complete pathological responses were obtained in patients with Hb values >13 g dl^−1^.

**Figure 1:**
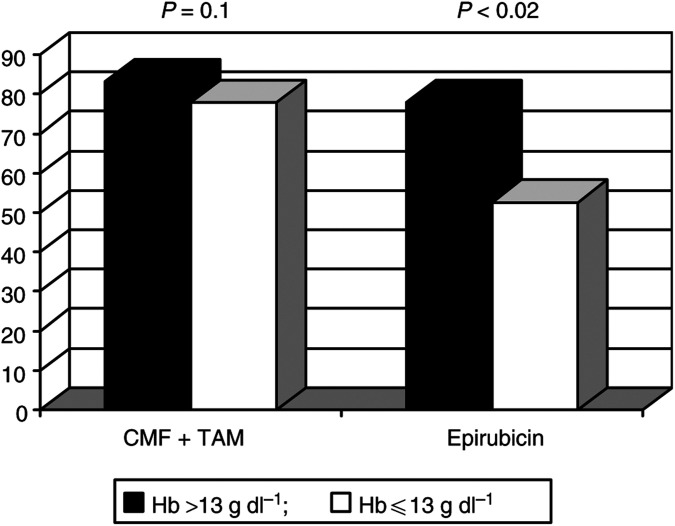
Distribution of disease response by baseline Hb status and treatment.

The predictive role of Hb status was more evident in the subset submitted to epirubicin than in the CMF-treated subgroup. In the latter group, only a trend (not significant) of greater response rate was observed in favour of baseline Hb >13 g dl^−1^ (Figure 1

).

In the multivariate analysis, Hb levels (considered as continuous variable) showed a significant independent role in predicting disease response after adjustment for menopausal status, T, N, c-erbB2, p53, bcl-2, ER, PgR, CD 34, Ki67, and treatment administered (Table 3

**Table 3:** Independent role of haemoglobin (Hb) in predicting disease response to chemotherapy according to multivariate logistic regression analysis

	**Hazard ratio**	**95% Confidence intervals**	***P***
T^a^	0.71	0.46–1.10	0.12
Node status^a^	1.08	0.49–2.37	0.84
p53^a^	0.70	0.27–1.83	0.47
Bcl-2^a^	1.24	0.41–3.73	0.70
Grading^a^	1.56	0.69–3.52	0.28
Ki67^a^	0.79	0.44–1.44	0.45
PgR^a^	0.72	0.31–1.67	0.44
ER^a^	1.03	0.28–3.70	0.96
Menopause^a^	1.20	0.53–2.73	0.65
Treatment	0.56	0.26–1.21	0.14
**c-erbB2^a^**	**2.58**	**0.93–7.16**	**0.05**
**Hb level^b^**	**1.48**	**1.03–2.11**	**0.03**

a These variables were categorised as displayed in Table 4.

b Haemoglobin level was introduced as a continuous variable.

).

At the last follow-up examination in May 2002, 44 patients (28.6%) progressed and 39 (25.3%) died, the median follow-up of surviving patients was 90 months (range 31–142). No difference in disease-free interval (median 130 months *vs* not attained, respectively) and overall survival (median not attained) was observed between the patients with baseline Hb<13 g dl^−1^ and those with baseline Hb >13 g dl^−1^.

### Relationship between Hb levels and patients and tumour characteristics

The variation in baseline Hb levels according to patient and tumour characteristics are depicted in Table 4

**Table 4:** Patient and tumour characteristics according to pretreatment haemoglobin (Hb) levels

	**Hb mean**	**s.d.**	***P***
Premenopausal	13.0	1.05	<0.01
Menopausal	13.6	1.02	

T2 tumours	13.4	1.13	n.s.
T3-4 tumours	13.6	0.89	

Node negative	13.4	1.13	n.s.
Node positive	13.4	1.00	

Grading 2	13.6	1.14	n.s.
Grading 3	13.4	1.03	

ER negative	13.4	1.16	n.s.
ER positive	13.4	1.05	

PgR negative	13.5	1.14	n.s.
PgR positive	13.4	1.03	

p53 negative	13.4	1.07	n.s.
p53 positive	13.5	1.06	

c-erbB2 negative	13.3	0.97	n.s.
c-erbB2 positive	13.6	1.25	

bcl2 negative	13.5	1.09	n.s.
bcl2 positive	13.4	1.07	

Ki67⩽10	13.5	1.13	
Ki67 11–29	13.3	1.01	n.s.
Ki67 ⩾30	13.7	1.12	

ER=oestrogen receptor; n.s.=not significant.

. Haemoblobin levels were significantly greater in postmenopausal patients than in premenopausal ones. No difference in Hb levels was observed stratifying patients according to tumour size, lymph-node involvement, tumour grade, steroid hormone receptor status, c-erbB2, p53, bcl2, and Ki67 expression.

**Figure 2:**
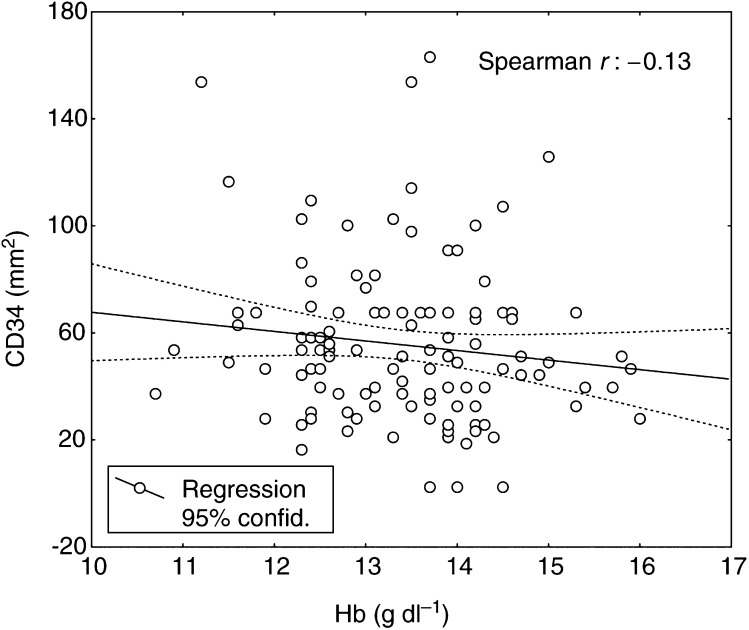
Relationship between intratumoral MVD and Hb values before chemotherapy.

No relationship was found between MVD and Hb values at baseline (Figure 2

); a very low relationship was found when considering Hb values and MVD at the end of treatment (Spearman *r*=−0.24, *P*<0.05).

Table 5

**Table 5:** Baseline and postoperative Ki67 expression and chemotherapy-induced Ki67 variation according to baseline haemoglobin (Hb) status

**Baseline Hb status**	**⩽13 mg dl^−1^**	**>13 mg dl^−1^**	**P**
Baseline Ki67			
Mean (s.d.)	16.8 (11.0)	17.9 (14.3)	n.s.
Median (range)	15.0 (1–50)	16.0 (1–90)	

Ki67 variation			
Mean (s.d.)	−4.1 (10.4)	−8.7 (14.2)	=0.04
Median (range)	−5.0 (−30–+40)	−5.0 (−87–+18)	

Postoperative Ki67			
Mean (s.d.)	12.6 (14.1)	8.8 (10.0)	<0.02
Median (range)	9.0 (0–80)	5.0 (0–50)	

s.d.=standard deviation; n.s.=not significant.

describes the changes in Ki67 expression before and after primary chemotherapy as well as the treatment-induced Ki67 variation, when patients were stratified according to baseline Hb status. The pretreatment Ki67 labelling index was the same for patients with low Hb levels and those with normal/high values. However, patients with Hb levels >13 g dl^−1^ showed a greater reduction in treatment-induced Ki67 expression and a lower Ki67 expression at postoperative evaluation than patients with Hb <13 g dl^−1^.

## DISCUSSION

In this single institution experience involving a relatively large number of patients with operable breast cancer, baseline Hb levels significantly influenced the tumour response to primary chemotherapy. This predictive role was also maintained after adjustment for disease stage, treatment, menopausal status, and several tumour characteristics in multivariate analysis.

The logistic regression analysis revealed that c-erbB2 expression was an independent predictor of overall disease response as well. c-erbB2 overexpression has been found to correlate negatively with the efficacy of CMF and TAM (but not the combination of these drugs) (Hamilton and Piccart, 2000). Conversely, a positive predictive value of c-erbB2 immunostaining has been found for anthracycline-based chemotherapy (Ravdin, 2001). The administration of epirubicin in more than half of our patients could account for the results obtained.

Anaemia is a common finding in cancer patients (Caro *et al*, 2001). This can be related to the disease process itself or its treatment, chemotherapy in particular (Groopman and Itri, 1999). In our series, however, the tumour itself was not the major cause of low Hb levels at baseline condition. Patients included in the study were substantially healthy females bearing relatively small primary tumours without metastatic disease. As shown in Table 4, Hb levels were significantly lower in premenopausal patients than in postmenopausal ones, suggesting that relative iron deficiency related to the menstrual cycle represented the most important mechanism. At surgery, the majority of patients had mild to moderate anaemia subsequent to chemotherapy. The moderate relationship between pre- and post-treatment Hb values suggests that postchemotherapy Hb is influenced by pretreatment values. As expected, the reduction in Hb was greater in the patient subset treated with epirubicin than in that treated with CMF. When patients were divided according to treatment regimen, the impact of Hb on disease response was greater in the subset treated with epirubicin than in the subgroup receiving CMF. Hypoxia-induced resistance of tumour cells to anthracyclines has been repeatedly described *in vitro* (Ikeda *et al*, 1999; Matthews *et al*, 2001). The generation under normal oxygenation of reactive oxygen species such as hydrogen peroxide and nitric oxide, which cause oxidative damage to cellular proteins and apoptosis, could be a possible mechanism (Ikeda *et al*, 1999; Matthews *et al*, 2001). In addition, in the absence of oxygen, P450 reductase interacts with anthracyclines, leading to cleavage of the anthracycline glycosidic bond and producing a molecule with no antitumour activity (Pan *et al*, 1981). In the CMF-treated group, most patients also received TAM concomitantly, which could have counterbalanced the cytotoxic resistance induced by hypoxia at least in part since the drug induces apoptosis that is ER mediated (Dorssers *et al*, 2001) and may not be influenced by tumour oxygenation.

It has been demonstrated that the oxygenation status of human tumours is independent of clinical tumour size stage and grade (Wouters *et al*, 2002). Our data agree with these findings; indeed, no relationship was found between Hb levels and these tumour characteristics. A wealth of laboratory data implicates hypoxia as a contributor to the malignant phenotype (Hockel and Vaupel, 2001). Our data did not show a relationship between Hb levels and either steroid receptor immunostaining or oncogene expression at baseline condition.

Tumour hypoxia has been found to stimulate tumour-induced neoangiogenesis (Hockel and Vaupel, 2001). Our data failed to demonstrate a relationship between Hb levels and the MVD. However, MVD is neither a measure of angiogenesis (Vermeulen *et al*, 2002) nor does it reflect the degree of perfusion of a tumour (Stohrer *et al*, 2000). Hence, the question of whether tumour hypoxia induced by low Hb values may contribute to the multifactorial stimulation of tumour-related neoangiogenesis could not be addressed by our study.

At least two mechanisms have been identified as being potentially responsible for chemotherapy resistance in anaemic patients, that is, the loss of apoptotic potential and the inhibition of cell proliferation. In this study, the apoptotic index was not measured; instead, proliferation activity was assessed by the expression of the Ki67 antigen. At baseline condition, no difference in the percent of Ki67-stained cells according to Hb status was found. After chemotherapy, however, reduction in Ki67 expression was greater in the patient subgroup with baseline Hb values >13 g dl^−1^ than in their counterparts, which produced lower KI67 immunostaining in postchemotherapy residual tumours. These data suggest that the antiproliferative activity of cytotoxic treatment is promoted by tumour oxygenation.

Residual tumour is, by implication, resistant to chemotherapy. Accordingly, our observation of greater Ki67 expression in the postoperative specimens of patients with low Hb levels may indicate the persistence of aggressive disease, with a higher risk of disease relapse.

No difference between disease-free survival and overall survival according to Hb status was observed, as the study was not powered enough to search for these differences. Interestingly, anaemia was found to have a negative predictive role for local RFS in a recent study involving 424 premenopausal patients receiving adjuvant chemotherapy (Sevelda and Gnant, 2002).

In conclusion, the study findings suggest that reduced Hb levels with resulting poorer tumour oxygenation could be implicated in the complex mechanisms of chemotherapy resistance of breast cancer. Whether treatment resistance in anaemic patients can be, at least partially, prevented or overcome by anaemia correction is a matter for further investigation.
